# Cyborgs in the Everyday: Masculinity and Biosensing Prostate Cancer

**DOI:** 10.1080/09505431.2015.1063597

**Published:** 2015-09-03

**Authors:** Gill Haddow, Emma King, Ian Kunkler, Duncan McLaren

**Affiliations:** ^a^ Science, Technology and Innovation Studies, The University of Edinburgh, Edinburgh, UK; ^b^ Midwifery and Allied Health Professionals Research Unit (NMAHP-RU), University of Stirling, Stirling, UK; ^c^ Edinburgh Cancer Centre, The University of Edinburgh, Edinburgh, UK

**Keywords:** in vivo biosensors, patient ambivalence, qualitative research, everyday cyborg, masculinity and stigmatisation

## Abstract

An *in vivo* biosensor is a technology in development that will assess the biological activity of cancers to individualise external beam radiotherapy. Inserting such technology into the human body creates cybernetic organisms; a cyborg that is a human–machine hybrid. There is a gap in knowledge relating to patient willingness to allow automated technology to be embedded and to become cyborg. There is little agreement around what makes a cyborg and less understanding of the variation in the cyborgisation process. Understanding the viewpoint of possible beneficiaries addresses such gaps. There are currently three versions of ‘cyborg’ in the literature (i) a critical feminist STS concept to destabilise power inherent in dualisms, (ii) an extreme version of the human/machine in science-fiction that emphasises the ‘man’ in human and (iii) a prediction of internal physiological adaptation required for future space exploration. Interview study findings with 12 men in remission from prostate cancer show a fourth version can be used to describe current and future sub-groups of the population; ‘everyday cyborgs'. For the everyday cyborg the masculine cyborg status found in the fictionalised human–machine related to issues of control of the cancer. This was preferred to the felt stigmatisation of being a ‘leaker and bleeder’. The willingness to become cyborg was matched with a having to get used to the everyday cyborg's technological adaptations and risks. It is crucial to explore the everyday cyborg's sometimes ambivalent viewpoint. The everyday cyborg thus adds the dimension of participant voice currently missing in existing cyborg literatures and imaginations.

## Introduction: The Development of *In vivo* Biosensors in a Cyborg Society

 … basically machines were not self-moving, self-designing, autonomous. They could not achieve mans's [sic] dream, only mock it … now we are not so sure. Our machines are disturbingly lively, and we ourselves frighteningly inert. (Haraway, 1991, p. 152)

Deaths from prostate cancer are predicted to exceed that from lung cancer by 2015 (Prior and Waxman, 2000; Scambler, 2009). Prostate cancer is the most common male cancer in Western societies. In the early stages of the disease, men usually present to their General Practitioner with urinary symptoms such as frequency, getting up during the night and a poor flow.

Prostate cancer is diagnosed from the results of a blood test. The ‘prostate-specific antigen’ (PSA) test measures the level of the antigen in asymptomatic men. It is thought a raised level of PSA is linked to prostate cancer; however, a positive result does not conclusively demonstrate it (Faulkner, 2012). Screening for prostate cancer is controversial in the views of some as,

[T]he PSA test has relatively high false positive and negative rates, prostate biopsies can miss cancers, and the highly variable nature of prostate cancer means there is potential for diagnosis of indolent cancers that may never present as a problem. (Clements *et al*., 2007, p. 8)

Nevertheless, if a PSA is taken and it is raised, a referral for a prostate biopsy may be made. From the patient's point of view then, there are generally no physiological symptoms to suggest that cancer may be present. We highlight the importance of this absence below.

Prostate cancer is an illness that some have suggested affects a masculine identity through for example, incontinence and impotence caused as a result of both illness and treatment (Conrad, 2007). Treatment options in the early stage of the disease when the cancer is localised can include periods of ‘watchful waiting’ when nothing is done until the disease progresses (Prior and Waxman, 2000). Most men however prefer to ‘do’ something with a treatment option (Chapple and Ziebland, 2002). Treatment might involve external beam radiotherapy that uses radiation to kill cancer cells. Quite often some patient's tumours demonstrate a resistance to radiotherapy. Many of the indicators of radiation response are found in the tissue and fluids immediately surrounding the tumour.

The development of biosensors is being undertaken in order to sense when the tumour is at its most vulnerable^1^ (Wilson and Gifford, 2005, p. 2389; Begg *et al*., 2011). Some have suggested that the monitoring of the tumour could take place when the patient is at home (Scarantino *et al*., 2002).^2^ The ability to supply wireless power and communications has already been demonstrated (Johannessen *et al*., 2004; Smith *et al*., 2007). The patient's treatment can then be scheduled to coincide with when the biosensor has indicated that the tumour is at its weakest. This has several benefits. Targeted radiotherapy is likely to have cost-saving implications given the optimal scheduling of radiotherapy delivery. It can also help minimise the side-effects that some patients suffer from such as dry mouth or mouth ulcers (in the case of head and neck cancers) to frozen shoulders and heart failures (in the case of breast cancer) (Begg *et al*., 2011).

A biosensor can be considered an active medical device as it is an instrument, which together with its software, can be used for diagnostic and therapeutic purposes, relying on a power source other than that generated by the body. The Active Implantable Medical Devices Directive (1993) (AIMD 90/385/EEC as amended by 2007/47/EC) defines an active medical implant as:

 … any active medical device which is intended to be totally or partially introduced, surgically or medically, in to the human body or by medical intervention in to a natural orifice, and which is intended to remain after the procedure.

We present findings that show men recovering from prostate cancer are extremely willing to have a biosensor inserted; to become cyborgs every day. Furthermore, participants assumed, indeed wanted, the biosensor to have a longer term functionality beyond that originally envisaged. This research speaks to the widening interest in theorising how technology is impacting on embodiment through a process of ‘cyborgisation’ (Gray, 1995a). Our participants did not self-identify as a cyborg. Yet, as a consequence of being permanently implanted and monitored, a cyborg status would be created for these men. Importantly, we describe this as creating an *everyday cyborg* status; a hybrid of machine and organism living in modern society.

Originally, the cyborg was used in an article to describe the bodily modifications required to create a homeostatic feedback system for ‘men’ to survive future space exploration (Clynes and Kline, 1960). This adaptation would not affect who the men were; changing neither their identity nor sexuality. The extreme versions depicted in the science-fiction of the cyborg, of the human–machine for example, are also generally male but the technology does affect their identity and crucially, their humanity. The human–machine is generally male, with traits of power, strength and control. The gendering of the cyborg is dealt with more critically in the science, technology and innovations studies (STIS) literature (Haraway, 1991; Hayles, 1995; Gray, 1995a, 2000, 2001, 2011, 2012) and in feminist STS literature (Penley *et al*., 1990; Kirkup *et al*., 1999; Henwood *et al*., 2001). In this STS literature, and taking the lead from Haraway (1991), technology is a means in which boundaries between animals and humans; the physical and the non-physical and animal–human/machines dissolved.

What the everyday cyborg adds to previous versions then is a recognition that a willingness to become cyborg is contextually dependent, for example, to avoid cancer. The present data suggest that prostate cancer threatens masculinity in a way that existence as a cyborg that is perceived as liberating and powerful, which offers action and control, does not. The stigmatisation of being a leaker and a bleeder for the men in our sample points to a cancer identity that victimises the patient as a person (Sontag, 1978). This experience influences the willingness to undergo a process of cyborgisation. But there is also ambivalence about the experiences of incorporating technology into the body and of becoming cyborg every day. A willingness to become and be a cyborg is also one that can be accompanied with ambivalence regarding the experiences of living as a machine–human. Also missing from previous accounts about space travel, or science fiction or liberation is a less imaginative concept of the everyday cyborg that outlines the risks involved in becoming cyborg. Such an account asks whether a person would want to become cyborg every day. What would the everyday cyborg say about the cyborgisation process? What would the benefits and challenges of living with implanted technologies be from an individual's viewpoint? For example, with automated technology brings with it risks of malfunction. And with implanted automation also comes the removal of autonomy from the individual.

Before outlining the results of the study and how the answers to these questions were reached, we briefly outline previous cyborg versions and why the term cyborg is used here. Particular attention is drawn to the reasons why individuals would want themselves and others to become cyborg, what an everyday cyborg actually is, and why this status carries with it its own unique risks. Hence, after outlining the findings we then reflect in the discussion about what the data might say to the cyborg literature as well as the argument that the process of cyborgisation is variable but inevitable at the level of the individual and society more generally (Gray, 2000).

## The Conceptual Work of the Cyborg

The term ‘cyborg’ was originally coined in the 1960s as a term to describe the mechanical adaptations to the body thought necessary to enable individuals to live in a hostile environment such as space: ‘For the exogenously extended organizational complex functioning as an integrated homeostatic system unconsciously, we propose the term “Cyborg”’ (Clynes and Kline, 1960). In the original usage of the term ‘cybernetic organism’ the cyborgs were referred to as men and how their bodies could be adapted. For the ‘cyborg-in-space’ it was essential to have an object, device or process which acted as a reactor so that any changes in the environment did not damage the internal normal physiological processes essential for survival. Key then to the original use of the concept is the regulation and surveillance of the body without the person necessarily being aware of it (Clynes and Kline, 1960 , p. 75). And yet, in Clyne's original conception of the cyborg, and others since then, these technological adaptations and implantations to the human body are seen as broadly acceptable, relatively risk-free and largely unproblematic for the person.

More well known is an extreme version of the cyborg. In the public imagination this cyborg is the science fiction image portrayed in books, films and other cultural forms (Oetler, 1995). Often these are images of beings who are human–machine hybrids that are stronger and more powerful, yet incapable of feeling and emotions. Therefore they are somehow less human and humane. Recent examples, are the Borg in Star Trek, the Cybermen in Dr Who, and the antagonists in films such as Robocop and Terminator (Goldberg, 1995). In an interview with Chris Gray, Clynes expressed horror at what his cyborg-in-space had become in the science fiction genre, ‘Well at first I was amused and then I was horrified because it was a total distortion … This recent film with this Terminator, with Schwarzenegger playing this thing-dehumanized the concept completely.’ The Terminator and others are examples of visible abominations of organic–inorganic machinations, and crucially, male and thus human–machine with emphasis on the ‘man’ in human.

The science-fiction cyborg is almost always invariably male, or asexual at best.^3^ This masculinity as well as inhumanity of the fiction cyborg is a trend that can be traced historically to the ‘creature’ created by a scientist ‘Frankenstein in the gothic novel by the author Mary Shelley' (Shelley, 1831 (1993)). In the introduction to the 1993 reprint, Jansson suggests:

For Mary Shelley, however, two of the most important aspects of science centre upon the essential ‘masculinity’ of scientific thought … This ‘masculinity’ is most evident in the removal of any feminine element from the Creature's ‘birth’; the scientific process activated by Victor excluded any sense of the humanity of the Creature. (p. x)

Although there may be some notable exceptions to the cyborg's masculine identity, these human–machines have the physical attributes of strength and power co-existent with the dominant Western ideals of masculinity (Connell, 1995).^4^ Gendering of the cyborg does not just prevail in our imaginations of the science-fiction or even in the ‘cyborg-in-space’ versions but explicitly with the technological focus and dominance found in war today. In, for example, ‘toys for the boys’ and in adaptions and control of the male penis for sexual attraction and intercourse (Gray, 2000). These extreme cyborgs in the imagination can be contrasted with the ‘mundane cyborg’ (Petersen, 2007; Mentor, 2011), where differences between the ordinary and the extraordinary arguably reflect questions of degree and not kind.

Another version of the cyborg emerged in feminist science studies and science and technology studies (STS). Here the cyborg has been deployed to challenge the dominance of dualisms inherent in gender for example. The feminist philosopher and social theorist Donna Haraway, in her seminal paper ‘The Cyborg Manifesto’ (1991) drew on the ‘cyborg’ to challenge longstanding dualisms between nature and technology, human and animal, male and female (Haraway, 2003). According to Haraway, the cyborg is a, ‘cybernetic organism, a hybrid of machine and organism, a creature of social reality as well as a creature of fiction’ (1991, p. 119). It is historical and also post-gender with an ability to liberate from classificatory categories. Not just a phantasm of fiction or the imagination but a ‘reality’ as a phenomenon that exists. Haraway deploys the cyborg as a positive feminist metaphor, a means of not only highlighting, but also, invalidating the inherent impurity of any dualistic system of thought or mode of being in the world. The cyborg for Haraway was our ontology and our existence. With technologies such as biosensors, a society of cyborg individuals implanted with technologies is being created. Furthermore, these everyday cyborgs are increasingly living in an environment dominated with technological relations of cyborg society. Yet the everyday cyborg is not necessarily an icon of liberation envisioned by authors, such as Haraway (1991, 2003) but, as we will show, may be a reflection of current technological developments and medical practices reifying existing inequalities in cyborg society.

At the time Haraway published her Cyborg Manifesto in 1985, many people were already living with technologically augmented bodies for medical therapy. Over 30 years later, medical technologies that augment and replace human organs and limbs have improved dramatically. Indeed, STS literature is beginning to acknowledge the experiential basis of cyborgisation for these patients (Bjorn and Markussen, 2013; Oudshoorn, 2015). Hayles (1995, p. 322) identifies the cyborg as existing in the technical sense; as being the 10% of the American public with ‘electronic pacemakers, artificial joints, drug implant systems, implanted corneal lenses, and artificial skin’. An even higher percentage, she sees as living as ‘metaphoric cyborgs’ with a life entwined in an information communication environment. For cyborg scholar Gray, the process of cyborgisation is akin to that of dying and death both sharing a variability but inevitability:

There are many different types and levels of cyborgization. The incorporated living elements (viral, bacterial, plant, insect, reptile, rodent, avian, mammal), the technological interventions (vaccination, machine prosthesis, genetic engineering, nanobot infection, xenotransplant) and the level of integration (mini, mega, mundane) can all vary, an infinite number of cyborgs, life multiplied by human invention and intervention. (2012, 29)

As society becomes invaded with information communication technology so too has the body itself. Yet the unevenness of this ‘invasion', charting who it is likely to affect and why, has not yet been well-documented or understood. Swallowing a pill or riding a bike does not make someone an everyday cyborg. Clynes suggested that ‘once you learn how to do these things automatically [ride a bike] the bike becomes almost a part of you’ (Gray, 1995a, p. 49). He termed this as becoming almost virtually a cyborg, but more of ‘a simple cyborg’. Then, to be an everyday cyborg, modifications are required that quite literally become part of a person and that are automated and beyond individual autonomy.

Technically, the possibility of creating cyborgs through the insertion of devices such as biosensors create their own unique challenges relating to the design of the technology in terms of size, power, accessibility, status and transference of information as well as the risks posed to the individual. Converging technologies such as biosensors can pose a number of problems as they draw upon the technologies of ‘informatization and miniaturization’ (Swierstra *et al*., 2009) bringing together biotechnology, nanotechnology and information technology. In doing so, the convergence may diminish the cogency of each function, creating additional risks not previously recognised. The biosensor will be able to transmit data about the tumour out of the body—that is, it will be able to send data to a monitor and a device programmer hence bringing into question the need for data security and accuracy.^5^

Given these challenges what then are the viewpoints of a group of possible beneficiaries, to having such a technology inserted into their bodies? To become cyborg every day. The cyborgs of extreme science fiction, of life in future space, or in feminist science studies, do not tackle what we tentatively refer to, as ‘everyday cyborg', of individual people willing to live with internal techno-mechanical modifications. By using the term ‘everyday’ we mean to (1) differentiate those living with technology from the other cyborg models of science-fiction, outer-space or critical STS and to (2) highlight that the ‘everyday’ is in itself an achievement and not something we can always take-for-granted (Das, 2010) To be an everyday cyborg builds on the previous literature about cyborgs (outlined above) insofar as (1) similar to the ‘in-space’ model, the technology is automated and implanted and the person may not always be aware of its function and functioning. The everyday cyborg is however (2) not monstrous as in the ‘sci-fiction portrayal’ but mundane in the everyday; someone who is not inhumane but is actually very human, (3) the person is a hybrid of physical and virtual convergences yet, is not an emancipatory political myth nor a positive feminist metaphor, but is part of a highly gendered biosocial phenomenon and (4) the everyday cyborg lives with risk and he is not in control of the process of cyborgisation, which is often created and mediated by experts.

## Methods

### Recruitment

We analyse the experience of cyborgisation and everyday cyborgs through the study of men recovering from prostate cancer. We purposively sampled for men based on their treatment status. As part of their radiotherapy they are fitted with fiducial gold markers that are inactive but cannot be removed due to the position of the prostate. This embodied knowledge of having objects implanted for therapeutic purpose and then left in the body was thought to produce points of view of the biosensor technology that was more ‘grounded’ and, importantly, based on actual experience rather than possible conjecture. This allows us to analyse the experiences of cyborgisation in an everyday context. The study recruited post-treatment prostate cancer patients from a Cancer Centre, at a UK National Health Service (NHS) hospital who were attending their follow-up clinic with a specialist nurse three months after the completion of successful external beam radiotherapy treatment. The specialist nurse approached participants at the review clinic. They explained the study to the participant giving them the participant information sheet and consent form. They asked for their consent to pass their contact details (name, e-mail address and telephone number) on to the social scientists.

Once the researchers received these details we contacted the participants enquiring about the participants’ continued interest and willingness to participate and also taking the opportunity to answer any further questions and reminding the participants that they were under no obligation to participate. If willing to be interviewed, a date, time and location convenient to them was arranged. Our intention was to interview the participants about their views of the intra tumour biosensor in light of their experiences of cancer but we did not want them to relive previous circumstances in an experiential and unduly upsetting manner. None of our participants demonstrated any visible distress or tiredness during the interview or in the wind-down time after. Full ethical clearance was gained from an NHS MREC (10/S1103/41). In the following accounts pseudonyms are used and care taken to avoid disclosing exact locations.

### Interviews

The interview was semi-structured with the ordering and form of the questions used flexibly although the main areas of interest were addressed around acceptability, visibility, willingness, control, ownership and information transference. Furthermore, we used vignettes and pictorial representations of the implants to stimulate discussion. Permission to record with a digital voice recorder was sought and none refused. The recordings were anonymised, transcribed and analysed; the latter with the aid of a computer-aided qualitative data analysis software Nvivo 9 to aid identification of key themes and to explore when issues were discussed in relation to each other (and if so, how regularly). These are part of standard qualitative analysis procedures (Glaser, 1965; Glaser and Strauss, 1967) and we used both an inductive and deductive approach to thematic development. For example, ‘getting used to’ was an important trope that arose *in vivo*, that is, in participant's own words.

### Sample

Of 15 participants initially contacted, 12 men were interviewed, with 4 carried out with wives present, occasionally prompting their partner's recollections or adding additional comments.^6^ Our participants were aged between 62 and 77 years, born between 1935 and 1950. All had been or were married and most, but not all, had children and grandchildren. Such demographic data provided context for the study; for example, who else was told about the diagnosis. The importance of demographics affecting views in a sample of this size cannot be established. We anticipated that being in recovery from cancer the participants might be more likely to offer positive views of the biosensor technology. Indeed, this appears to be the case, and was commented on by the participants themselves. However, we were also struck by the range of ambivalences also voiced, impressing on us that views were not overly determined by this experience; experience which by necessity we were keen to identify and include in the first place.

## Findings: Everyday Cyborgs

As is often the case, prostate cancer was ‘symptom less’. Participants told us they had sought advice from their GP regarding other health problems such as not sleeping (Mr Williams), bladder infection (Mr Dean), deafness (Mr Dubont) and, ‘that problem of getting up in the night’ (Mr Shane). For many, the cancer diagnosis came as unexpected: Mr Nairn said, ‘I feel well, I've always been well, even before surgery, after surgery and ever since. There's only blood tests suggested I had prostate cancer, so you have to take people's word for it.’ The uncertain and asymptomatic nature of the disease, the unpleasantness of procedures involved in attempting to establish a diagnosis, as well as the perceived stigmatised position that prostate cancer sufferers occupy in society was commented upon. Mr Williams, summed it up, at the very beginning of his interview:

I went to the doctor to discuss not sleeping and he said we'd better do a blood test because we don't know, and that was that. I really forgot about it and the next thing I was asked to go to the urology at the hospital. I didn't phone the doctor, I just went up there thinking ‘why am I here?’, ‘I'm perfectly fit’. I cycled over, I sat down and there were all these poor men as I saw them, I know there *are vulgar terms for them*, ‘*leakers’* and ‘*bleeders’* or whatever, but I didn't regard myself as part of that at all … I remember having an image of myself being in that room floating above what was going on down below as I was duly fingered and all the usual sort of things. (Mr Williams, emphasis added)

With little warning, Mr Williams became a member of the ‘leakers’ and ‘bleeders’ subjected to intrusive tests and procedures. A common treatment for the cancer was external beam radiotherapy with most participants demonstrating clear understanding of the process:

The preparation for radiotherapy starts with a CT scan to identify, it was prostate cancer I had/have, and they plant gold [fiducial markers] seeds in three positions in the prostate and a CT scan identifies where it is so they can target exactly where it's going. They put marks on your body so they can line that up and get the accuracy correct then it homes in on that. All they do is expose, they put tattoo marks on your body, just a black spot, which they line up and target the beam on that. (Mr Scot)

Targeted radiotherapy consisted of a few minutes of treatment occurring five days a week over a period of months. The process was described succinctly by Mr Melrose as, ‘taking longer to get your stuff off and get on the table [than] to get the treatment'. As mentioned in the above quotation, ‘gold seeds’ or fiducial markers are used so that the radiotherapy can be administered with the greatest possible accuracy. The participants that had received external radiotherapy all had markers in their bodies. Few admitted hardly ever giving any thought to them. Jokes were made during the interview about the fact they were made of gold and their perceived cost. Mr Dalkeith said, ‘ … they just told me they were a couple of wee gold markers and I thought it's probably the best bit of jewellery I've got'. These men may be partly physical/non-physical with their ‘wee gold markers’. At this point, the men are not yet cyborg in the cyborg-in-space or cyborg-in-fiction version of the term. Neither do they appear to represent a blurring of Haraway's (1991) boundaries between the animal–human (organism) and machine although they are technically hybrid. Control of their physiological processes or organs are not yet mechanised, automated nor automatic and they are not yet human–machine. The conditions which will make the possibility of becoming a cyborg every day has however been set.

### Would You Have One?

We moved from discussing the treatment that was offered to patients to more ‘hypothetical’ scenarios, whereby *in vivo* biosensors might be offered as a future option. Few participants had heard the term ‘biosensor’ before. Those that had could not remember where they might have come across it. Most were willing to accept the sensor for a variety of reasons including trust in medical science but mainly influenced by the desire to avoid future cancer:

Mr Burgh: Frankly I do as I'm told. My knowledge of medicine and the likes is virtually nil, so if the doctor tells me I need something I do it, it's as simple as that. It's in my own interests as well so it's very likely I wouldn't have a problem with it.

GH: Is there absolutely anything at all that we've talked about or perhaps not talked about that would put you off (the in-vivo biosensor) or that you want to add?

Mr Cole: As I said, I had such a fright I would have gone through anything.

Cyborgisation is then a process that is variable and contextually dependent based on the reasons for the technology being implanted. In these cases, cancer was an important factor. However, the medical professional's influence was also key and appears to be uncritically accepted suggesting that the everyday experience of becoming cyborgs is mediated by experts. Willingness to incorporate technologically advanced and automated electrical parts is not a decision the individual would freely take. It is one that the everyday cyborg may have to accept.

### More Than Planned

It became apparent in interviews that some of the participants assumed the biosensors could be a continual part of their recovery. A cyborg status, if embraced, was not seen as temporary one but would remain a permanent feature of their life. That is, biosensors would continually monitor the prostate, not just at a time when radiotherapy would be given, but also as a long-term warning system. The biosensor is the technological adaptation, the feedback mechanism envisaged by Clynes and Kline (1960) in their ‘cyborg-in-space’ version, surveying the body automatically without the person ever being aware of it. Although it is unclear whether the adaptions for space exploration would be permanent, for cancer protection and the everyday cyborg it was. Mr Williams continued:

 … if I had a bio-sensor in me and there was no-one could read it I wouldn't fuss about whether I could turn it on or off or not. For me these are irrelevant questions, it's the concept of, right from the beginning, if I'd been asked, we might want to put a bio-sensor into your body and it might just have to stay there forever and it might only work for six months, would that be alright or would you be happier, obviously you would be happier with one that worked forever. It seems to me that would be a, the concept of it not working hadn't occurred to me … 

Hence, as we went onto discuss in the interviews, ethical questions around data security were felt generally to be ‘irrelevant’ for the everyday cyborg. Reiterating positive views about the technology, and the professionals involved, Mr Cole had a laissez-faire attitude to data sharing, ‘I feel if you're being treated then so long as it's the professionals involved in it, I don't give a monkeys who knows.’ The ability for the biosensor to transmit data from the prostate to an external monitor about the receptivity of the tumour to external beam therapy was relatively well received over all.

For some participants in the current sample what appeared more important, than data security was it (the biosensor) ‘working forever’. This ‘forever functionality’ would be welcome, as Mr Scot suggested; ‘I would think that's right because if you think of the PSA, which is a blood test, if the PSA can be monitored by a biosensor then that's a big step forward.’ Mr Shane went further. He suggested that those who had cancer ‘in the family’ could be offered biosensors:

This possibly could be given to people who have a history of, because I think it's genetic, I think there's a lot, I'm sure they know that if you have a father or brother, anybody that could be given this device then presumably this could be monitored that way rather than the biopsy before it gets to the stage when you have to get a biopsy.

In this account, the everyday cyborg is now far removed from the abstract concept of a critical STIS cyborg (Haraway, 1991). Although the cyborg modifications are liberating in that there is a form of surveillance and protection from cancer it is constraining insofar as it is only preferable to the cancer patient alternative. Furthermore, this hybridity is not emancipatory of the power in the dualisms that so often mark contemporary society. The cyborg is a family man and he is vulnerable to the recurrence of disease and humane in his dealings with significant others. The everyday cyborg is not then science-fiction's monster and although he is male, he advocates an interesting version of ‘cyborg creep’. In current accounts, everyday cyborgs embrace promissory technologies protecting their own bodies but also the bodies of those important to them. Everyday cyborgs care.

Such long term and extended surveillance of the prostate by biosensing is not an intended functionality of the biosensor as currently envisaged by developers. A possible drug delivery system is being discussed however, and this was well received by the participants, again tying in with the regulation aspect of Clynes and Kline (1960) original cyborg-in-space model. Everyday cyborgs therefore would benefit from further convergences in the technology that would not only sense but maintain the physiological status through a feedback mechanism reacting to change. This type of automation enhances the health of the everyday cyborg freeing the individual from having to, for example, give themselves daily injections. It is not so dissimilar from the sci-fi human–machines who benefit from automated functioning (Goldberg, 1995; Gray, 1995a). The everyday cyborg benefits also and would hardly be aware of the drug being delivered. Their hybridity is both active and quiet:

GH: You might want to use it to deliver a drug as well.

Mr Williams: That would be a good thing. I've got a friend who is diabetic and if he was given something that was implanted, however crudely on his body, rather than having to jab the thing every day, he would go for it, so it's all very detail-specific.

EK: If it was going to release a drug, would you prefer it if the doctors could tell it when to release drugs or that the bio-sensor would be smart enough to do it when it thinks it needs to?

Mr Lamb: If it's been made by doctors who know what they're doing the probability is that the bio-sensor is smart enough to know when to release it so, whatever.

Cyborgisation of the body, produced through the insertion of automation, is mediated through the medical system and embraced by the participants. Becoming cyborg is not seen as becoming monstrous as in the science-fiction version (Gray, 1995b). Although hybrid, the hybridity is a necessity for the everyday cyborg. In a sense, it is not that far removed from future space-travel (Clynes and Kline, 1960). But for Mr Lamb, there were additional risks to having automated treatments such as a drug release system in the body needed in order to survive:

I think you'd have to make the assumption that whatever was contained within the thing was almost certainly going to be used. Because if suddenly they said, gosh, the tumour has died, we don't need to release this, do you mind spending the rest of your life with this extraordinary drug in your body that might leak out? (Mr Lamb)

Some participants were simultaneously incredulous and apprehensive about the unintended consequences of automated drug delivery:

I think we're getting into the realms of the *science fiction* now. If the thing could administer drugs, which is another great idea, but when would it know it was giving the right drug at the right time? And also if, say for example, after getting so much of the drug and the tumour changed, would the same drug have a detrimental effect on it or would you think that you're putting this thing in and you're leaving it to do the work of what you would say the oncologist, or would you have to keep monitoring it in MRI scans? (Mr Melrose; emphasis added)

Cyborgisation is risky for the individual. More commonly, contraceptive implants for women are regularly used in the UK and can be inserted in the upper arm slowly releasing progesterone. Contraceptive implants can be removed and their malfunctioning, as technology is wont to do, may not have fatal consequences. If the biosensor malfunctions or if the drug leaks then the repercussions are far more likely to be significant in terms of having a negative impact on the everyday cyborg's health. For the everyday cyborg automated medicine is one aspect of convergence functioning that may carry additional benefits regarding ability to survive in a hostile environment whether it be in space or with cancer. However, further and increasing convergences of technology, especially of automated treatment, carries risks not commented on previously. Neither the in-space cyborg (Kline and Clynes, 1960) nor the feminist science studies literature (Haraway, 1991) discusses the possibility of the technology malfunctioning when it is in control. Haraway (1991) famously suggested she might rather be a cyborg than a goddess but the former may come with hidden costs. The cyborg is technology in the body; a hybrid indeed but both these two entities remain materially intact. The signification of the cyborg is different to its materialisation granted but being a cyborg can be detrimental to your health.

### Risks: Space and Time and is It Mine?

As discussed above, Mr Melrose was suspicious of the automated treatment potential of biosensors as with automation there is the risk of malfunction. Yet Mr Melrose had a laissez-faire attitude to the removal of the sensor as long as it was not having any detrimental effects. He suggested that leaving the device in:

It wouldn't worry me if it [bio-sensor] couldn't be removed and it wasn't going to be causing any after-effects, fair enough, that would be alright, there's no problem there. Because after all they're putting these markers in and they're leaving them.

Likewise, Mr Burgh, ‘The seeds are going to be there anyway, so keep that company’. The biosensors are artificial in form as are the fiducial marker seeds. The former however have an additional active functionality understood by the participants.

Then the everyday cyborg is not a simple cyborg insofar as it was defined by Clynes in his interview with Gray (1995b, p. 40). Although Clynes suggested that when the person is able to automatically accomplish tasks such as riding a bike that technology virtually makes them a simple cyborg (1995, p. 49) the bicycle does not actually become a part of the everyday cyborg in the way that an implanted machine quite literally does. Taking a pill may mean a temporary addition to the body but it does not become permanent part of the body in the way a device does; it is not an automated permanent fixture for example. Indeed, Mr Shaver said ‘If it [biosensor] did its job it would be part of me’. As alien in matter as the technology is, it can become acclimatised to and incorporated into the organic body. The cyborg's technological adaptations are felt then to be no different to their (non) awareness of other organs. The everyday cyborg becomes a hybrid of the organic-mechanical. And this hybridity is not extra-ordinary or monstrous but becomes ordinary and mundane. A person cannot physically touch inside their own body handling the major organs, and indeed the organs themselves may be (or become) alien to the person.^7^ In the same way as we do not own our organs most participants asked felt that they did not have ownership of the biosensor. It was felt to belong to the UK NHS. Parallels were drawn with other implantable medical technologies such as cardiac pacemakers:

Mr Melrose: Before it's in your body then obviously it belongs to the National Health Service.

GH: When it's in your body?

Mr Melrose: I would still think it belongs to them, although it's in your body it's only there to help you get better hopefully.

GH: What about when it's no longer working and it's still in your body?

Mr Melrose: If it's no longer working and they can't take it out then fair enough. But if it's in there to stay then it's in there to stay, you've got something of the National Health Services.

GH: Would you not feel it's yours?

Mr Melrose: *When you say it's yours there's nothing you can do with it. It's not as though you can take it out and play with it or sell it.* It's no advantage other than the thing is in you. It's like having a pacemaker, they put the pacemaker in you and it's in there while you're alive but once you die they've got to take that out (emphasis added).

Given the participants did not feel that they owned the sensor but it was part of them in the same way their own organs might be part of them, we wondered whether control would be an important aspect for them—an area touched on above but warranting further exploration. The biosensor, as a mechanical device, would be controllable by others, by experts, in a way that human organs are not for example.

The reason why researching people's views of implantable medical technologies is important, is because of the intimacy of the machine in relation to the corporeal. Ironically, this bodily intimacy also implies distance in terms of personal control. Being implanted with automated technology has detrimental consequences in terms of control and autonomy for the everyday cyborg. Nowhere does this seem to appear in alternative versions and models of the cyborg. With implantable devices although they may in some respects become parts of the body, as unknowable to the person as their other organs, the implants still maintain a connection to others. The biosensor belongs to the NHS. The implications of this ownership and, importantly, control by others for the cyborg in science-fiction have been explored (Goldberg, 1995, p. 233) however not for any of the other versions and certainly not, until now, for the everyday cyborg.

Given the possible risks associated with automated drug delivery for example, several control options were offered regarding whether the biosensor could be switched on or off (e.g. by the patient or a medical professional). By far the most popular response was for the sensor to have automatic control functioning similar in the way that internal organs function. Part of the reason related to patients’ unwillingness to take ‘the controls’ of the biosensor was you could ‘forget’ it (Mr Melrose) or bemusement, ‘why would I, as a patient, be wanting to switch it on and off?’ (Mr Nairn) and finally, to not having to explain the sensor to people. Mr Shaver explained:

I just think it would be better all-round, better for me not having to carry something around or lose it or explain it to people. I didn't tell the family I had prostate cancer until I started my treatment because I didn't want the kids, the young ones, looking at me differently, and I didn't want them all crying on me, so I bit the bullet and got a telling-off later on. (emphasis added)

Contrast this unwillingness to take personal control of the sensor with a discussion with Mr Cole about whether members of the general public would be willing to have a biosensor:

Mr Cole: … Although a lot of people might say ‘I don't want to walk about with something stuck in me’, I think that would be an issue for some folk. For me, quite a long list of folk that I've known who have died of cancer, anything to avoid that, so I *would put up with that* but a lot of folk might think, ‘I don't want somebody else *controlling my bits and pieces’*. That could well be an issue. (emphasis added)

Unwillingness to take personal control of the device compared with unwillingness to allow others to control the biosensor suggests autonomy is heavily context dependent on the device functions and functioning. It is importantly variable in the process of cyborgisation at the individual and societal level. Put simply, in terms of machines and technology working inside the body, who is controlling what, for whom and why?

### Why Take the Risks in Cyborgisation?

Risk is contextually dependent on issues of control but views of it are affected by whether there is felt to be a need to take the risk. Any type of invasive surgery brings with it the risk of additional traumas that can range in the severity of consequences. The chances of the biosensor causing the patient damage through infection on implantation, to blood clotting or embolism is a possibility, although slim ones. We discussed these risks with all participants and whether this would change their views about biosensor acceptability. Whereas the risk of infection was seen as acceptable it was not the case when it came to discussing possible fatalities from embolisms.

Made explicit in some accounts was a ‘weighing up', of the possibility of a fatality from the biosensor against the severity of the cancer. For Mr Nairn,

[T]hat is a more difficult one to answer because I find myself in the situation that I'm quite well at the moment. Prostate cancer as far as I'm aware can progress slowly therefore I might prefer to delay something with a risk until a time that is, where the need is more pressing.

Mr Shaver linked it to his age saying he, ‘would accept it [the risk of embolism] if I was this age, yes. I wouldn't like it maybe but I'd accept it’. Iatrogenic effects of a cyborg adaption were assessed in relation to the severity of the cancer and the age of the person (Conrad, 2007). Technology, control and risk perceptions are weighed up in terms of costs and benefits—How old am I? How sick am I? Am I worth it? If I am worth it, is the device worth the risk?

Generally the process of cyborgisation was worth the risk. The most salient feature of all the interviews in the current research was the context of the participants having survived cancer. The impact that this had on the views in relation to the discussion of biosensors cannot be understated or ignored and is a key factor influencing positive attitudes. As Mr Nairn put it at the end of his interview:

Again it comes back to the fact that any medical treatment comes with a risk and it's a case of balancing risks, and with cancer the risk of the disease is worse than the risk of what could go wrong’ [And much later] ‘I'm intrigued by you actually need to speak to people about it because I would have thought people would *be willing to try anything* if it might reduce the threat. (emphasis added)

Such sentiments fuel discussions around the robustness of consent both as a practice and value and points to the importance of examining the motivations of participation and the social context in which views are expressed. Access by others to the biosensor and of possible infections caused by its presence are risks for the everyday cyborg they are prepared to take. They live in the shadow of cancer and there is a universal willingness to ‘try anything’.

Yet, in this case, to have a biosensor implanted and to become cyborg in the everyday was not whole-heartedly embraced. It was one thing to be willing to be implanted but it was another to live with it. The stoicism found in the discourse of acceptance emerged as a theme relating to a ‘getting used to’ trope.^8^ When considering, for example, the scenario whether the implant might need to break through the skin barrier in order to access an external power source, this initially provoked negativity from the participants.

The visibility of the adaption by breaking through the skin barrier, was the least liked aspect of having a biosensor implanted. This poses questions about cyborg identification by self and others and is worth exploring further. Is it because the internal adaption can be seen? One can see that there is something inside in a way that an internal organ cannot? Yet, this discomfort with having wires through the skin, was also often accompanied alongside a ‘getting used to’ statement. Mr Cole ‘I had a catheter in for months and you get used to that, I think you would probably get used to it [having visible wires]. Again, if you've got cancer I think I would put up with anything.’ For the everyday cyborg it is preferable for what goes on inside with their biosensor to stay inside. Perhaps to some extent, this echoes the visible cyborg identity that the science-fiction model presents (Goldberg, 1995). The science-fiction cyborg's modifications are visible and their inhumanness therefore readily apparent; for the everyday cyborg such close associations with the monstrous are not welcomed. They are not inhumane, on the contrary, their everyday status makes them even more humane. Associations with the science-fiction cyborg are visible when wires are coming through the human skin reminding the everyday cyborg of their hybridity. And yet, is something else the everyday cyborg *can* get used to. As Mr Scot suggested ‘Put it this way, if having a biosensor with wires poking out through my skin for a limited period of time it was going to do me good, I would have it’. Mr Nairn commented, ‘It's the difference between what I would like and what I will accept’. Mr Dean saw it as,

I think anything that's going to help you in a situation like that is very acceptable, and it's only for, in my case it was seven weeks. *It's not the end of the world is it*, if it's actually doing good or helping. (emphasis added)

Indeed, evident in most interviews was a view summed up my Mr Lamb's response, when asked whether it would have troubled him if he had needed brachytherapy. ‘When you get cancer anything's better than death’ he said.

## Conclusion

In this paper various versions of cyborgs are discussed. The cyborgs in popular imagination abound in literature and in the media (in-space and in-fiction) and are versions which emphasise masculinity and traits such as power, strength and control. Such traits are not shared by the everyday cyborg who may be male, as in the science-fiction model, but was often a family man. His cyborg presence in the everyday is one that can present risks to him but are set against the background of life itself. Willingness to becoming cyborg, living with technological adaptations that protect his body and the bodies of others, has more appeal than the lack of control over bodily functions that having prostate cancer, or perhaps any cancer has. It is preferred even when he has no autonomy over the automated device that others, such as experts, might have. And when they have data about his physiology.

It is not clear however whether the willingness to become cyborg was related specifically to having had cancer which was often mentioned, or whether it was having recovered from prostate cancer specifically. The latter is a form of cancer that threatens masculinity both in terms of its consequences and treatment. It is difficult to say whether the stoicism that was expressed in living as an everyday cyborg was related to this as being a possibly negative alternative.

The experience of cyborgisation was tempered with reflections of what living with technological adaptations would mean. Specific risks around technological malfunction were mentioned and biosensors were internal adaptions that were balanced against other risks, ‘When you get cancer anything's better than death’. Hence, the importance and frequency of ‘getting used to it’. The term ‘getting used to’ is commonly used in the English language describing the process of becoming familiar or accustomed with. This might be a process of acclimatisation that is particularly gendered. It is a stoicism certainly in this research relating to men supposedly having superior strength and power to carry on in the light of adversity.

There is the opportunity to explore from the point of view of beneficiaries what becoming cyborg means for them in terms of the benefits and challenges to their existence as embodied individuals. A cyborg ontology, as espoused by critical feminist studies, requires a method of social enquiry that reaches into the experiential and relational, enquiring about the viewpoint of possible beneficiaries. One such account is given here that focuses on the material repercussions of the cyborgisation process. Or rather the personal effects of living as cyborg in the everyday. Other research is required for recipients who have medical devices such as implantable cardiac defibrillators and, interestingly, who also tend to be male (Cunningham *et al*., 2011). Comparative research on biosensors with women who are recovering from breast cancer, would further increase understanding of the gendering of willingness to become cyborg and the ambivalence and stoicism that may follow. This may be the case regarding the ambivalence around the need for ‘acclimatisation’ when technology is inserted into specific bodily locations associated with a male or female sexuality identity.

The internal modification of the human body through technologies, such as biosensors, is akin to the regulative and surveillance implants in the original cyborg-in-space insofar as becoming cyborg is about the technological automation of human physiological processes and organs. The implantable machines have a level of functionality and autonomy. Technologies such as implantable cardiac defibrillators and pacemakers, cochlear implants and deep brain stimulators, are contributing to a society of cyborgs. More of us will live as everyday cyborgs, because as we live longer the quality and quantity of implantable technologies that repair or replace the functions of the body will become increasingly common. This is at the same time as the ability of the cyborg adaptations become increasingly sophisticated. Ironically, the more intimate these technological automatic adaptations are to the inside of the body resulting in the cyborg—the more the autonomy of the cyborg is challenged. The biosensor, in some cases, becomes a part of the person in a similar way that a human organ does; both unknowable. Yet it is a technology that can malfunction. It has also been created and man-made. One that can be controlled by experts in a way that a person's organ may not be.

What the everyday cyborg adds to previous versions then is a recognition that a willingness to become cyborg is contextually dependent, for example, to avoid cancer. The present data suggest that prostate cancer threatens masculinity in a way that existence as a cyborg that can be liberating and powerful, insofar as stigmatisation of being a leaker and a bleeder for the men in this sample, is avoided (Sontag, 1978). This experience influences the willingness to undergo cyborgisation. But there is also ambivalence about the experiences of incorporating technology into the body and of becoming cyborg. A willingness to become cyborg is also one that can be accompanied with ambivalence regarding the experiences of living as a machine–human hybrid. Missing from previous accounts about the cyborg in future space travel, or in science fiction and public imaginations or liberations from the power dualisms in feminist science studies, is a less imaginative concept of the everyday cyborg. And yet answering questions, for example, about whether a person would want to become cyborg points to the challenges of having to deal with a hybridity that might compromise personal autonomy. Despite not wanting to take controls of the biosensor participants are aware of the risk of the biosensor being out of control. Ironically, a reason for becoming cyborg in this sample, was to overcome a lack of control over malfunctioning organic body processes.

Creating different types of cyborgs—machines in the human—poses different types of risks to the individual dependent on the functioning of the technology. Or rather, dependent on the malfunctioning of the technology. Increasing the number of cyborgs in society also carries with it challenges. How the actual technology itself is socially stratified in the population and whether some groups are more likely (or not) to become, everyday cyborg is key. Liberation does not come without a cost both at the individual and social level. Where does this challenge re-situate the cyborg in the feminist science studies literature? The implications of this research are that the creation of everyday cyborgs is reifying existing gender stereotypes and inequalities because the dualisms of technology and human remain in the hybridity that is the everyday cyborg.

## Notes

1 Radiation therapy is required for the treatment of over 50% of patients with cancer and has been argued by some to be highly effective in reducing deaths Delaney *et al*. (2005, pp. 1129–1137).

2 Indeed, technologies such as heart pacemakers or implantable cardiac devices can transmit data about the condition of the heart from within the patient's body to a monitor that the patient keeps at home. The monitor then transmits the data to a central repository at the clinic or hospital that health professionals can access via a secure intranet Halperin *et al*. (2008, pp. 30–39).

3 The exception in popular media, it could be argued, is the overtly sexualised female cyborg whose technological modifications focus on female traits such as the ability to listen or female reproductive anatomy (Six Million Dollar Woman and Seven of Nine in Star Trek).

4 The modern cyborg can be a male soldier with a power masculinised and militarised for a technological war. The male reproductive organ, the embodiment of masculinity has been the subject of technological interventions ensuring that control over its functioning can be depended upon (quoted in Gray, 2000).

5 For example, pacemakers and ICDs can malfunction and send data that is erroneous Halperin *et al*. (2008, pp. 30–39), Gould *et al.* (2006, pp. 1907–1911). Halperin and colleagues were able to access private information and interfere with the integrity of an implantable cardiac defibrillator for example Halperin *et al*. (2008, pp. 30–39).

6 Of the three that did not take part this was due to inability to re-contact one, another did not feel fully recovered and the last did not want to take part.

7 For an interesting discussion of how cancer tumours can themselves be seen as ‘alien’ see Dixon-Woods (2007).

8 In Fujita *et al*. (2006) study of hip replacements patient's reported a period of having to ‘get used’ to their hip replacement, often due to the differences in the environment between home and hospital (e.g. no hand rails). Some reported the effects of the hip on their body perception, feeling that they did not like having something artificial inside them. Others also reported a sense of loss about their original hip and even though it did not work properly it was still ‘theirs’ Fujita *et al*. (2006, pp. 81–87).
